# IUC24390-83 Morphology of circulating tumor cells: how it might relate to biology and clinics in prostate cancer

**DOI:** 10.1093/oncolo/oyaf248.014

**Published:** 2025-08-29

**Authors:** R Wenta, J Richert, A Muchlinska, B Pieczynska-Uzieblo, K Miszewski, M Matuszewski, A J Zaczek, N Bednarz-Knoll

**Affiliations:** Laboratory of Translational Oncology, Medical University of Gdańsk, Poland; Laboratory of Translational Oncology, Medical University of Gdańsk, Poland; Laboratory of Translational Oncology, Medical University of Gdańsk, Poland; Laboratory of Translational Oncology, Medical University of Gdańsk, Poland; Department of Urology, Medical University of Gdańsk, Poland; Department of Urology, Medical University of Gdańsk, Poland; Laboratory of Translational Oncology, Medical University of Gdańsk, Poland; Laboratory of Translational Oncology, Medical University of Gdańsk, Poland

## Abstract

**Background:**

This study aimed to characterize measurable morphological features such as size, shape, presence of protrusions, and micronuclei in circulating tumor cells (CTCs) in prostate cancer (PCa) patients and assess their clinical relevance.

**Methods:**

Peripheral blood (PB) and tumor-draining vein blood (TDVB) samples were collected from 118 PCa patients at the Medical University of Gdańsk between 2018 and 2023. CTCs were phenotyped using epithelial (pan-keratins, K) and mesenchymal (vimentin, V) markers, with exclusion of non-tumor cells using CD45, CD31, αSMA, and CD29 markers. Imaging flow cytometry and QuPath software were used for the acquisition of CTC images, phenotypic and morphological analysis. Statistical analysis was performed with IBM SPSS Statistics. Representative images of CTCs were shared via CTC Atlas (www.CTCAtlas.org).

**Results:**

A total of 1,437 CTCs in TDVB and 578 in PB were identified across 4 phenotypes: K + V−, K + V+, K − V+, and K − V−. CTCs were larger than leukocytes, with K + V− and K − V− cells being the largest, and K − V+ the smallest. Epithelial-to-mesenchymal transition (EMT) CTCs were significantly smaller in PB. Cytoplasmic protrusions were detected in a small subpopulation of CTCs. When detected in TDVB-derived CTCs, protrusions were significantly more frequent in epithelial CTCs compared to EMT-CTCs (*P* < .001). Micronuclei were observed in < 1% of CTCs in TDVB (limited to K + V− and K + V+), increasing to 3% in epithelial CTCs in PB. Small CTCs were associated with the shorter time to biochemical recurrence (*P* = .041, HR 2.198, CI95% 1.034-4.669). PB-derived CTCs were significantly smaller (*P* < .0001) and more irregular (*P* < .0001) in patients with newly diagnosed metastatic PCa (*n* = 13) than in those with localized disease (*n* = 556) (median diameter: 9.9 µm, range 7-23 µm vs 16.5 µm, range 7-62 µm; circularity: 0.67 vs 0.76). Smaller CTCs detected in TDVB and/or PB indicated patients’ shorter time-to-biochemical recurrence (*P* = .041, HR 2.198, CI95% 1.034-4.669).

**Conclusions:**

CTCs display morphological heterogeneity across phenotypes. This is the first report to quantify EMT-CTC morphology and identify features such as protrusions and micronuclei. Smaller and morphologically irregular CTCs appear to correlate with PCa aggressiveness and poorer clinical outcomes, suggesting that even such simple CTC characteristics might identify CTCs associated with disease progression.

